# Circulating Endothelial Microparticles Reduce in Concentration Following an Exercise Programme in Women With Polycystic Ovary Syndrome

**DOI:** 10.3389/fendo.2019.00200

**Published:** 2019-03-29

**Authors:** Richard J. Kirk, Leigh A. Madden, Daniel J. Peart, Myint M. Aye, Stephen L. Atkin, Rebecca V. Vince

**Affiliations:** ^1^Sport, Health and Exercise Science, Faculty of Health Sciences, University of Hull, Kingston upon Hull, United Kingdom; ^2^Centre of Biomedical Research, Faculty of Health Sciences, University of Hull, Kingston upon Hull, United Kingdom; ^3^Sport, Exercise and Rehabilitation, Faculty of Health and Life Sciences, Northumbria University, Newcastle upon Tyne, United Kingdom; ^4^Faculty of Health Sciences, Hull York Medical School, University of Hull, Kingston upon Hull, United Kingdom; ^5^Weill Cornell Medical College, Al Rayyan, Qatar; ^6^Royal College of Surgeons Ireland, Al Sayh, Bahrain

**Keywords:** polycystic ovary syndrome, endothelial microparticles (EMPs), exercise, endothelial function, obesity

## Abstract

**Purpose:** Endothelial dysfunction is a known comorbidity in women with polycystic ovary syndrome (PCOS). The aim was to assess if supervised, moderate intensity exercise could potentially impact markers of endothelial disruption; endothelial cell derived microparticles (EMP).

**Methods:** The current study investigated the effects of a supervised 8-week moderate intensity exercise programme on EMP in women with PCOS (*n* = 11) and control women free from any known disease (*n* = 10). EMP were enumerated via specific antibody (CD105, CD106) labeling and flow cytometry.

**Results:** CD105+MP significantly reduced in women with PCOS from pre to post-exercise programme, with CD105+ MP reducing from 2114 CD105+ MP per μl platelet free plasma (PFP) to 424 CD105+ MP per μl PFP (*p* = 0.025). Control women showed no significant change in CD105+ MP (*p* = 0.25) after completing the same exercise programme. CD106+ MP showed no change in either PCOS (*p* = 0.95) or control groups (*p* = 0.99). No significant correlations existed with the changes in EMP compared to body composition changes as a result of exercise.

**Conclusion:** Supervised, moderate intensity exercise independent of substantial weight loss reduced circulating CD105+ MP, likely reflecting an improvement in endothelial function in women with PCOS compared to healthy control women. Additionally, EMP may be a useful marker for physical improvement in exercise programmes for clinical populations.

## Introduction

Polycystic ovary syndrome (PCOS) is one of the most common endocrine disorders in women of reproductive age (1) and can account for up to 18% of the population depending on the diagnostic criteria used (2–4). PCOS represents a significant burden on the healthcare system due to its exhaustive list of related complications (2). Women with PCOS are known to have increased intrinsic insulin resistance (IR) when compared with non-PCOS women, independent of obesity (1, 5). PCOS is also known to increase the prevalence and risk of cardiovascular disorders including hypertension and dyslipidemia and infertility (4, 6). Furthermore, it is noted that between 40 to 60% of PCOS women are either overweight [body mass index (BMI) > 25 kg^.^m^2^] or obese (BMI > 30 kg^.^m^2^) with increased central adiposity (7). The main risk factors for development of PCOS manifest as atypical precocious puberty, premature pubarche, obesity, and metabolic syndromes in childhood (8, 9).

One abnormality that presents in PCOS women is endothelial dysfunction (ED) the underlying mechanism of which remains unclear but may be related to the hypertension and dyslipidemia that is reported (10, 11). ED has been assessed using various methods in women of this population, such as through flow-mediated dilation (FMD) (12–15) and EndoPAT-2000 (16). In addition a relationship between ED as measured by FMD and insulin resistance was shown previously in children and adolescents (17). Endothelial adhesion markers have also been studied in PCOS women (10, 16, 18). Diamanti-Kandarakis et al. (10) studied 25 women with PCOS and 25 women without PCOS investigating ED by measuring endothelial activation markers, as well as brachial artery FMD (BA-FMD). FMD values were significantly lower in the PCOS group, as well as ET-1 levels. Also, the PCOS group had higher levels of high sensitivity C-reactive protein, soluble intercellular adhesion molcule-1 (sICAM-1) and soluble CD106 (VCAM-1) compared to the control group, suggesting that PCOS women do present with an abnormal endothelial status, and endothelial adhesion markers can predict this altered state. Given that PCOS is associated with an increased cardiovascular disease (CVD) risk, and reduced endothelial functioning increases this risk (18), the clinical importance of lifestyle improvement on endothelial function in PCOS needs investigation.

Incorporating exercise as a treatment for PCOS may be favorable considering the benefits that exercise has in other conditions that are associated with PCOS (19). Lifestyle modification has been endorsed by the Androgen Excess and PCOS Society as a primary choice in the prevention of CVD (4). There are well-established benefits of exercise and weight loss in women with PCOS on factors such as reproductive function (20) and improvements in CVD risk markers such as obesity and cardiorespiratory fitness (21), but there is limited data on the effect of exercise on EF in women with PCOS (18). Exercise training improves EF in individuals presenting with similar risk factors to PCOS women, such as patients with type 2 diabetes mellitus (22) and hypertension (23).

Endothelial microparticles (EMP) offer an insight into the state of the endothelium and are known to be elevated in diseases characterized by ED (24, 25). EMP have also been shown to increase after exercise/endothelial stress in healthy individuals (26, 27) but their characterization over a longer period in response to exercise training in disease states associated with ED remains relatively novel. Markers previously used to identify EMP include CD105 (Endoglin), a constitutively expressed endothelial cell marker and also markers of endothelial cell activation such as CD106 (vascular cell adhesion molecule-1). Willis et al. (28) has previously shown an increased percentage of Annexin V positive MP in the blood of women with PCOS compared to controls, although EMP were noted as infrequent using a CD144 antibody for detection.

We hypothesized that EMPs would be disordered in PCOS reflecting the ED and that exercise may ameliorate those changes. The aim of this study was to assess ED utilizing CD105+ MP and CD106+ MP in women with PCOS undergoing a supervised 8-week moderate intensity exercise programme and control women free from any known disease. The aim was to assess if this type of exercise could improve EF in this population, and if there was a relationship between EMP and other factors, such as body composition and cardiorespiratory fitness.

## Patients and Methods

### Outline

Favorable ethical opinion for the study was gained through the NHS (LREC reference: 10/H1313/44). All of the participants recruited for this study were aged between 18 and 40 years of age. Pre-menopausal PCOS women were recruited who had been outpatients at the Hull and East Yorkshire Diabetes, Endocrinology and Metabolism Clinic in the past 5 years and were eligible if they showed characteristics of PCOS as described by the Rotterdam consensus (29). PCOS was diagnosed based on the presence of oligomenorrhoea, clinical or biochemical hyperandrogenism, and polycystic ovaries on ultrasound (after exclusion of other endocrine causes of hyperandrogenism). All non-PCOS women had regular menses and no evidence of clinical or biochemical hyperandrogenism. Any volunteer suffering from an existing medical condition that was defined as a contraindication to exercise or injury was excluded from the study. At each initial visit, participants were screened via a pre-exercise medical questionnaire to highlight any contraindications to the test protocol and written informed consent was provided following successful completion of the inclusion [aged 18–40, premenopausal women with PCOS (as defined by Rotterdam consensus) and exclusion criteria (regular exercise three times a week for last 3 months, pregnancy/breast feeding/attempting to conceive, history of cardiovascular, renal, hepatic and thyroid disease, history of diabetes mellitus, history of physical disability to exercise, history of allergy to insulin/soy oil/purified egg (intralipid), currently on oral anti-diabetic medicine improving insulin sensitivity, family history of sudden death]. BMI categories were defined as in the ACSM guidelines (2003) and those between 18.5 and 40.0 kg/m^2^ were eligible.

### Participant Characteristics

Fourteen women with PCOS (mean ± SD, age 28.75 ± 6.61 years, BMI 30.70 ± 6.02 kg/m^2^) and 11 matched controls (age 23.74 ± 6.11 years, BMI 25.92 ± 5.39 kg/m^2^) volunteered for this study and all gave written informed consent. All of these women were successfully screened and eligible for entry into the study, and they then began the 8-week exercise programme. The end of the exercise programme was deemed as 8 successful weeks, and subsequently the final assessment session occurred (post). Only those participants who managed to successfully finish the 8-week exercise programme were included in the data analysis. Participants were discontinued from the study if they became pregnant, missed more than 50% (6 sessions) of exercise per month, if the participant had to use any concomitant medications detailed in the exclusion criteria during the study period, or if her consultant physician or general practitioner requested that the participant should be withdrawn from the study in their best interest. Four women were unable to fully complete the exercise programme and were not entered into the final analysis as a result. Reasons for discontinuation of the study were personal problems (2, both PCOS group), pregnancy (1, PCOS group), and lack of attendance (1, non-PCOS group). The final participants analyzed were those that successfully completed the 8-week exercise programme, which consisted of 21 women (11 PCOS (age 28.00 ± 6.72 years, BMI 31.15 ± 6.30 kg/m^2^) and 10 controls (age 24.26 ± 6.18 years, BMI 25.92 ± 5.39 kg/m^2^). Glucose disposal rates were calculated as previously described utilizing hyperinsulinaemic euglycaemic clamps (30) at baseline and after completion of the programme.

### Baseline Assessment

Eligible participants for the study attended the Michael White Diabetes Center, Hull, UK within 1 week of the baseline assessment visit (pre), which took place at the University of Hull. At this initial visit, fasting venous blood samples were taken and analyzed immediately for the measurement of CD105+ MP and CD106+ MP. Within 1 week of this visit, participants arrived at the University of Hull where they completed a pre-exercise questionnaire. Participants were instructed to abstain from alcohol, caffeine, and exercise 24 h prior to the testing, and all baseline assessments took place between 08:30 and 10:00 in order to maintain consistency amongst participants and to reduce the effect of any circadian variation in biomarkers, which have been shown to change over a 24 h period (31). Once appropriate screening had taken place and eligibility for the exercise programme had been confirmed, anthropometric measures were recorded. Immediately after this, participants performed a ramped maximal exercise test (VO_2max_) in order to determine the individuals target workload for the exercise sessions.

### Exercise Training Programme

Within a week following the baseline assessment, participants began attending 3 supervised exercise sessions per week for a period of 8 weeks. Where possible, each session was 1 h in duration depending on their ability to complete the sessions with no complications. The programme used either a Woodway ELG55 motorized treadmill (Woodway, Weil am rhein, Germany), or a HP Cosmos Pulsar Treadmill (H/P/Cosmos) with the same protocol. Participants performed all sessions on a motorized treadmill working at or as closely as possible to 60% VO_2max._ VO_2_/kg was measured after the warm up, which lasted for 5 min at 4.5 km^.^hr^−1^ and for a period of 10 min in order to confirm the appropriate exercise intensity. The intensity of exercise was then adjusted by altering the speed of the treadmill if this value was not within ±2.5% of the target oxygen uptake. Following this 10 min gas collection, the facemask was withdrawn with the speed of the treadmill remaining as it was. A further gas collection was made at 40 min to confirm the desired intensity for a 5 min period. If this intensity was out of range then the treadmill speed was once again altered if required. Heart rate (HR) and rate of perceived exertion (RPE) (32) were monitored every 15 min throughout the session. If participants felt that they could not continue with the exercise for reasons such as injury or fatigue, they were able to stop at any time if necessary. Likewise, if it meant reducing the intensity for a period of time in order for them to recover then this was permitted, otherwise the intensity remained at the level pre-determined. Each session ended with a 5 min cool down at 4.5 km^.^h^−1^ and participants would then be free to leave once HR returned to within 120% of basal levels. The participants in the study were not asked to alter their diet in any way and were to continue as normal with their calorie consumption throughout the exercise programme. During exercise session visits, fluid intake was permitted *ad libitum*.

### Mid-Point Assessment

Participants performed an exact repeat of the baseline assessment within 3 days after the 4 successful weeks of the exercise programme had been completed. This mid-point assessment also aided in altering the prescribed exercise intensity for the exercise programme sessions thereafter if the individual managed to improve their fitness over the 4 weeks of initial training.

### Final Assessment

This final assessment at the end of the exercise programme for each participant was a repeat of the baseline and mid-point assessments.

### Venous Blood Samples

Venous blood samples were drawn from the antecubital vein via a standard venepuncture procedure into Vacuette blood tubes (Vacuette®, Greiner BIO-one, UK). Blood samples were taken no longer than 1 week prior to baseline assessment (pre), then after 4 successful weeks of the exercise programme (mid), and again in the final week no more than 1 week after ceasing with the exercise programme (post). Venous samples were all taken at or as closely as possible to 09:00 h following an overnight fast.

### Endothelial Microparticles

**Figure 1:**
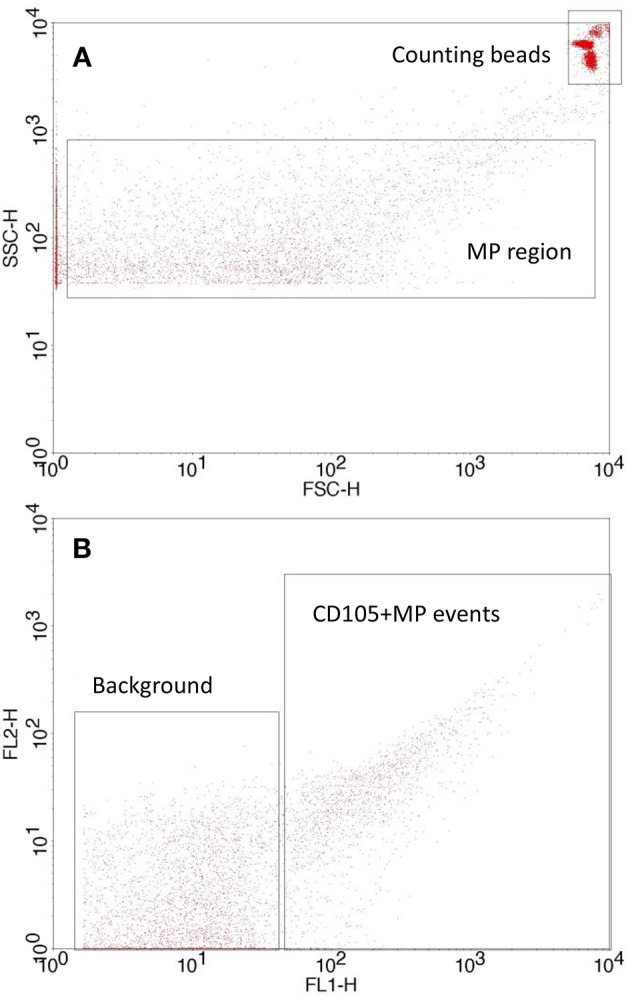
Flow cytometry setup showing **(A)** MP and counting bead gate on a forward scatter/side scatter plot with a side scatter threshold set according to the megamix protocol and **(B)** typical antibody labeled MP events vs. background/unlabeled events. MP are quantified using the counting beads.

Citrated blood was analyzed by obtaining platelet rich plasma through centrifugation (180 g, 10 min) using a Heraeus Labofuge 400R Centrifuge (Kendro Laboratory products, UK), which was then removed carefully into a 1.5 ml polypropylene tube. Platelets were removed by further centrifugation (12,000 g, 10 min) using a HeraeusBiofuge Pico Centrifuge (Kendro Laboratory products). Subsequently, samples of platelet free plasma (PFP) (25 μl) were incubated with 4 μl of either IgG1 CD105:FITC conjugate (AbDSerotec, UK) or IgG1 CD106:FITC conjugate (AbDSerotec) in the dark at room temperature for 30 min. Quantification was achieved by adding filtered phosphate buffered saline (150 μl PBS; 0.01 M phosphate buffer, 0.0027 M KCl, 0.137 M NaCl, pH 7.4 and autoclaved; Sigma, UK) and Accucheck counting beads (25 μl, Invitrogen, UK) immediately prior to analysis by flow cytometry (BDFACSCalibur®). A MP region (0.2–0.5 μm, Figure 1A) was set using megamix beads (Biocytex, France) according to an established protocol (33) and 25,000 events were counted for MP analysis, with positive MP being defined as events with an increased mean fluorescence intensity (Figure 1B), and were quantified in relation to counting beads according to manufacturers' instructions.

### Statistical Analysis

Central tendency and dispersion of the sample data are represented as the mean ± SD. Any changes in biochemical markers across condition and time were analyzed using linear mixed models. The change in anthropometric measurements across the exercise programme within the two populations were investigated using paired samples *t*-tests with Sidak adjusted *p*-values to control for family wise type I error rate. Relationships between EMP and variables were analyzed using Pearson Correlation Coefficients. Two-tailed statistical significance was accepted at *p* < 0.05.

## Results

### Baseline Characteristics

Table 1 displays the baseline characteristics of control and PCOS women. There was a significant difference between the groups in systolic blood pressure (SBP) (*p* = 0.027), which was higher in the PCOS group. VO_2max_ was significantly higher in the control group (*p* < 0.001). Waist to hip ratio (WHR) (*p* = 0.019) and waist circumference (WC) (*p* = 0.033) were significantly higher in the PCOS group when compared to control women. Levels of CD105+ MP were also significantly elevated in women with PCOS (*p* = 0.021).

**Table 1:** Baseline characteristics between control and PCOS women.

**Variables**	**Control baseline**	**PCOS baseline**	***P*-value**
	**(*n* = 10)**	**(*n* = 11)**	
Age (years)	24.26 ± 6.18	28.00 ± 6.72	0.202
Body mass (kg)	71.04 ± 16.42	85.45 ± 18.91	0.080
SBP (mmHg)	123.00 ± 11.44	132.36 ± 11.47	0.027^a^
DBP (mmHg)	77.50 ± 9.30	81.82 ± 11.21	0.290
VO_2_max (ml^.^min^−1.^kg^−1^)	36.26 ± 6.38	26.32 ± 4.63	<0.001^a^
BMI (kg^.^m^2^)	25.92 ± 5.39	31.15 ± 6.30	0.056
WHR	0.79 ± 0.07	0.86 ± 0.06	0.019^a^
WC (cm)	83.01 ± 14.20	98.05 ± 16.35	0.033
Glucose disposal (mg/kg min^−1^)	4.80 ± 1.73	3.26 ± 0.80	0.016
CD105 + MP (per μl PFP)	388.2 ± 113.5	2,113.9 ± 951.7	0.021^a^
CD106 + MP (per μl PFP)	7,165.1 ± 4403.8	7,625.6 ± 5,427.8	0.942

*Body mass, SBP, DBP, VO_2max_, BMI, WHR, WC, CD105+ MP and CD106+ MP are compared at baseline between the two groups*.

a *Significantly different between groups (p < 0.05). All data represented as mean ± SD, except for CD105+ MP and CD106+ MP which are represented as mean ± SEM*.

### Effect of Exercise on Measured Variables

Table 2 displays the changes in biomarkers as a result of the exercise programme in control and PCOS women. Body mass was unchanged in control women, but those with PCOS managed to significantly reduce their body mass at mid exercise programme (*p* = 0.027) and post-exercise programme (*p* = 0.027) when compared to pre exercise programme. Body mass in PCOS women decreased from baseline from 85.45 kg to 84.45 kg (−1.2%) at mid exercise, and then 84.04 kg (−1.7%) once the exercise programme was completed. SBP improved pre to mid (*p* = 0.031), pre to post (*p* = 0.002), and mid to post (*p* = 0.024) study in PCOS women. Diastolic blood pressure (DBP) also improved pre to post (*p* = 0.020) and mid to post (*p* = 0.005) study in this group. There were also improvements in VO_2max_ values from pre to post study, with values rising from 26.32 to 29.71 ml^.^min^−1.^kg^−1^ (*p* = 0.015) and also mid to post, from 26.43 to 29.71 ml^.^min^−1.^kg^−1^ (*p* = 0.002) in the PCOS group, while BMI was improved from pre to post (*p* = 0.028) and pre to mid (*p* = 0.015) point in the study. There was a decrease in WC in this population from mid to post-exercise intervention, decreasing from 96.97 to 96.12 cm (*p* = 0.035).

**Table 2:** Clinical characteristics of the control and PCOS women at baseline, mid, and post 8 weeks of aerobic exercise training. Body mass, SBP, DBP, VO_2max_, BMI, WHR and WC are compared between both groups.

	**Control (*****n*** **=** **10)**			**PCOS (*****n*** **=** **11)**		
**Variables**	**Pre**	**Mid**	**Post**	**Pre**	**Mid**	**Post**
Body mass (kg)	71.04 ± 16.42	70.66 ± 16.19	70.11 ± 15.83	85.45 ± 18.91	84.45 ± 19.02^a^	84.04 ± 19.54^a^
SBP (mmHg)	123.00 ± 11.44	120.50 ± 10.28	117.50 ± 7.65^a^	132.36 ± 11.47	128.73 ± 11.03^a^	124.91± 10.40^a^^b^
DBP (mmHg)	77.50 ± 9.30	75.00 ± 6.82	73.10 ± 5.43	81.82 ± 11.21	81.36 ± 12.36	76.64 ± 8.82^a^^b^
VO_2_max (ml^.^min^−1.^kg^−1^)	36.26 ± 6.38	37.49 ± 6.96	39.21 ± 5.82^a^	26.32 ± 4.63	26.43 ± 4.09	29.71 ± 5.32^a^^b^
BMI (kg^.^m^2^)	25.92 ± 5.39	25.78 ± 5.36	25.58 ± 5.18	31.15 ± 6.30	30.79 ± 6.32^a^	30.69 ± 6.48^a^
WHR	0.79 ± 0.07	0.78 ± 0.06	0.79 ± 0.07	0.86 ± 0.06	0.85 ± 0.07	0.85 ± 0.06
WC (cm)	83.01 ± 14.20	82.41 ± 14.19	82.16 ± 14.57	98.05 ± 16.35	96.97 ± 17.90	96.12 ± 14.67^b^

*Data are represented as mean ± SD*.

a *Significantly different compared to pre (p < 0.05)*.

b *Significantly different compared to mid (p < 0.05)*.

The control population only showed significant improvements in SBP pre to post-intervention, decreasing from 123 to 117.5 mmHg (*p* = 0.035) and VO_2max_ pre to post-intervention (36.26–39.21 ml^.^min^−1.^kg^−1^, respectively, *p* = 0.017). No other significant change was observed in any other measured variable in this group.

### CD105+ MP

**Figure 2:**
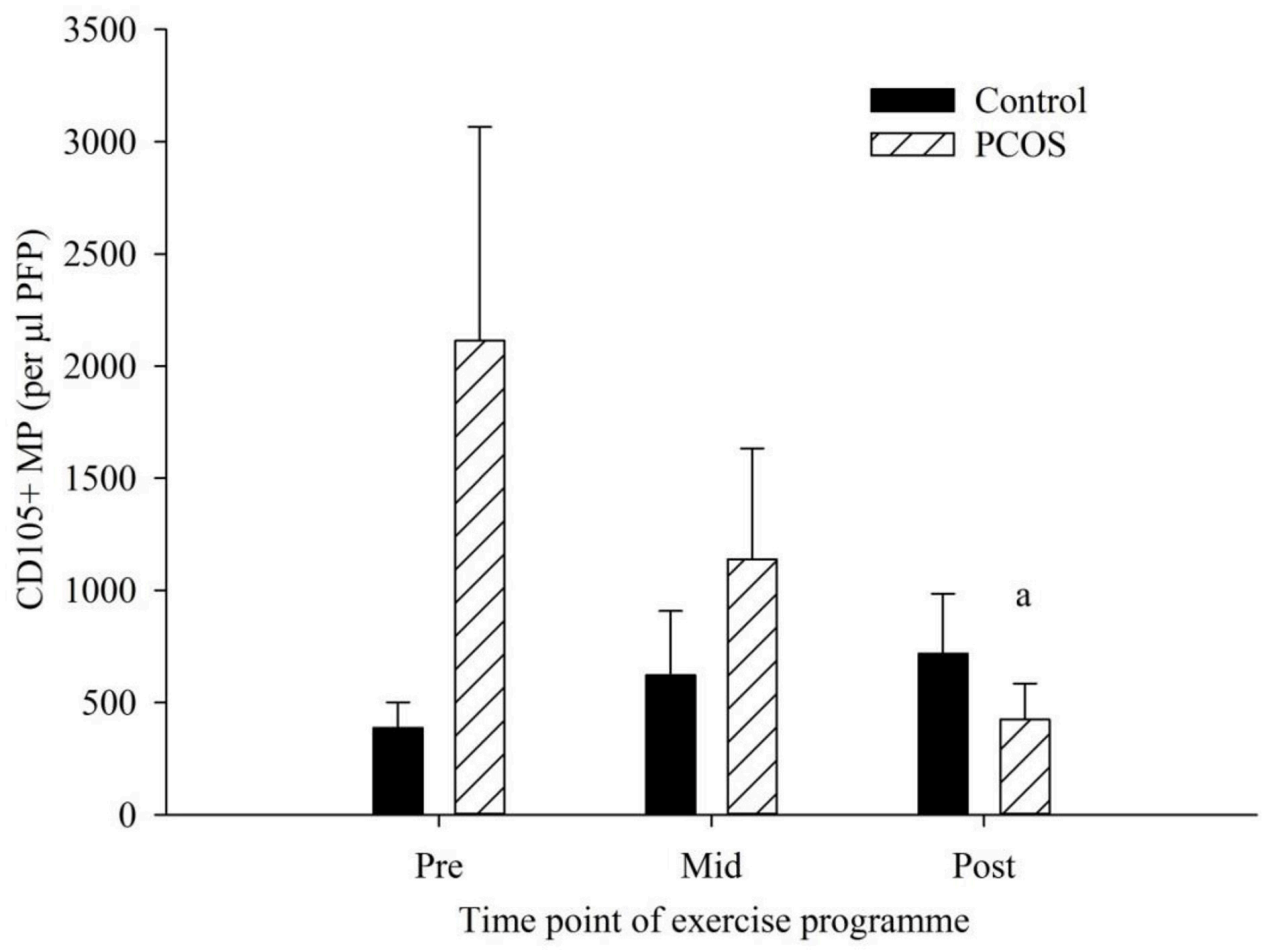
CD105+ MP in control (dark bars) and PCOS (hatched bars) participants at pre, mid, and post-exercise intervention. Data represented as the concentration of CD105 + MP per μl PFP (means ± SEM). ^a^Significantly different compared to pre in PCOS group (*p* = 0.025). Error bars represent mean ± SEM.

EMP were enumerated by flow cytometry (Figure 1). The PCOS group were the only group to see any significant changes in blood variables (Figure 2), showing significant decreases in CD105+ MP from pre to post-exercise intervention (p = 0.025). The number of MP decreased from 2114 CD105+ MP per μl PFP to 424 CD105+ MP per μl PFP. The control group conversely showed little change over the exercise programme, and no significant difference was observed pre to post-intervention (*p* = 0.25).

### CD106+ MP

**Figure 3:**
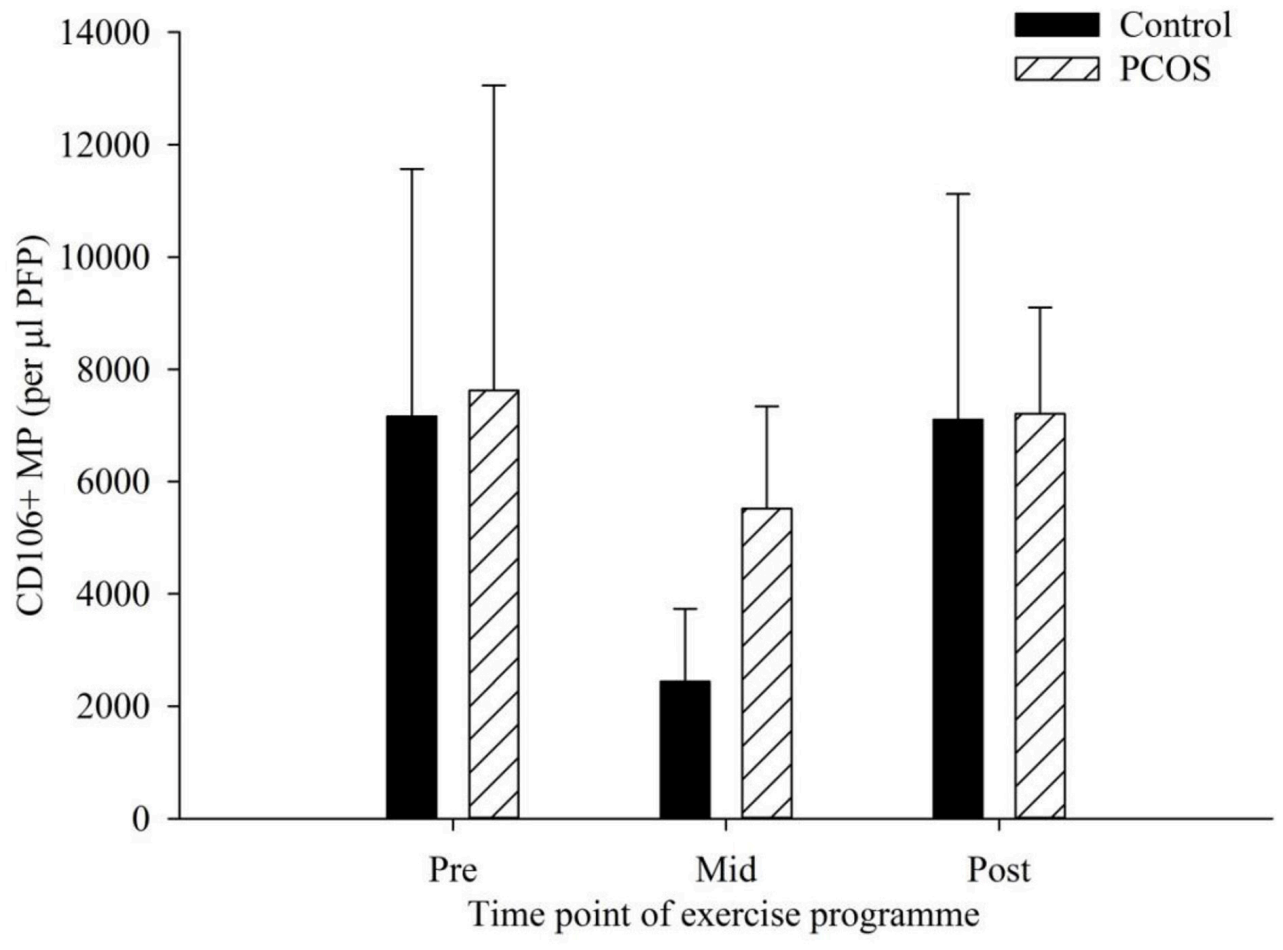
CD106+ MP in control (dark bars) and PCOS (hatched bars) participants at pre, mid, and post-exercise intervention. Data represented as the concentration of CD106+ MP per μl PFP (means ± SEM). Error bars represent mean ± SEM.

There were no significant differences in CD106+ MP as a result of exercise at any of the time points measured (Figure 3). The PCOS group showed a very minimal decline from pre to post-exercise intervention, with levels of CD106+ MP falling from 7,626 to 7,210 CD106+ MP per μl PFP (*p* = 0.95). Similarly, the control groups mean CD106+ MP levels changed from 7,165 to 7,105 CD106+ MP pre to post-exercise intervention (*p* = 0.99). Interestingly, there was a decrease, albeit not significant, in the control group from pre (7,165 CD106+ MP per μl PFP) to mid exercise programme (2438.9 CD106+ MP per μl PFP, *p* = 0.35), with values returning to basal values by the end of the programme. There was also a slight decrease in the PCOS group from pre to mid exercise programme, with CD106+ MP decreasing from 7,625 to 5,516 CD106+ MP per μl PFP (*p* = 0.71), with values returning to basal values by the end of the programme.

### Correlations of Anthropometric Data With EMP Data

Table 3 shows the correlations of CD105+ MP and CD106+ MP with the variables measured throughout the exercise programme in all PCOS women and control women. There were no significant correlations with EMP data and any of the measured variables presented in Table 3 for either group, suggesting that any changes in EMP were independent of these variables. The strongest correlation occurred between SBP and CD105+ MP in PCOS individuals, with suggestion that the higher the SBP the greater the amount of CD105+ MP, but this was not significant (*p* = 0.092).

**Table 3:** Correlation of CD105+ microparticles (MP) and CD106+ MP with variables measured in control and PCOS participants throughout the entire exercise programme from pre, mid to post 8 week intervention; *r* value determined from Pearson's Correlation Coefficient using group means ± SD, except for CD105+ MP, and CD106+ MP (mean ± SEM).

**Variable**	**Control** ***n*** **=** **10**		**PCOS** ***n*** **=** **11**	
**CD105+MP**	***r-*****value**	***P-*****value**	***r*****-value**	***P*****-value**
Body mass (kg)	−0.119	0.531	−0.041	0.822
SBP (mmHg)	−0.067	0.724	0.303	0.092
DBP (mmHg)	−0.050	0.794	0.193	0.289
VO_2max_ (ml^.^min^−1.^kg^−1^)	0.136	0.481	−0.094	0.613
BMI (kg^.^m^2^)	−0.117	0.537	−0.008	0.966
WHR	0.027	0.891	−0.090	0.648
WC (cm)	0.051	0.789	0.125	0.528
**CD106+** **MP**	***r*****-value**	***P*****-value**	***r*****-value**	***P*****-value**
Body mass (kg)	−0.202	0.284	0.020	0.915
SBP (mmHg)	−0.131	0.490	0.176	0.337
DBP (mmHg)	−0.211	0.263	0.022	0.904
VO_2max_ (ml^.^min^−1.^kg^−1^)	−0.014	0.944	0.114	0.543
BMI (kg^.^m^2^)	−0.186	0.324	−0.017	0.929
WHR	−0.236	0.218	−0.165	0.402
WC (cm)	−0.249	0.185	−0.074	0.708

### Insulin Resistance

**Figure 4:**
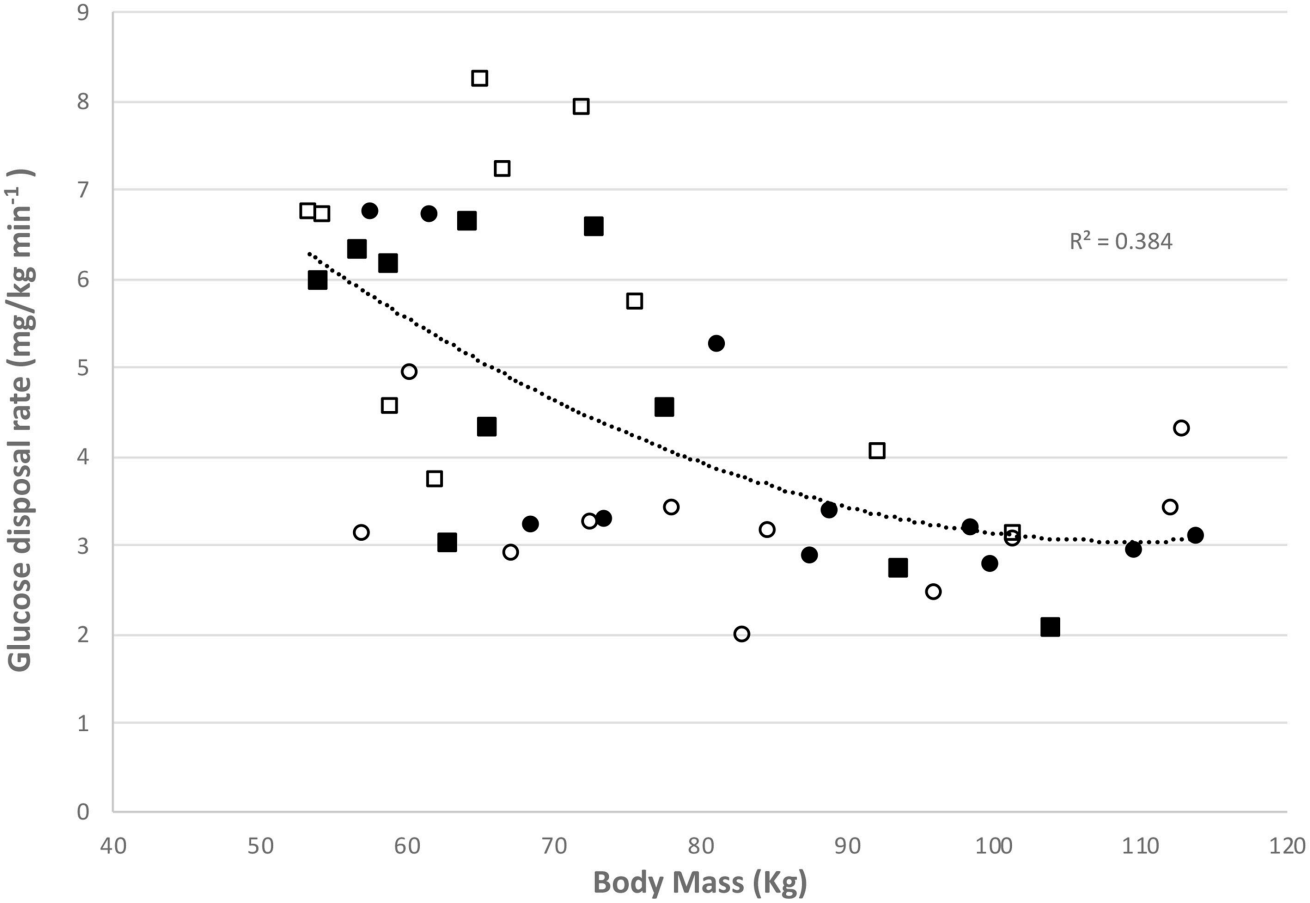
Relationship between glucose disposal rate and body mass in controls at baseline (■) and at final assessment (□) and women with PCOS at baseline (∙) and at final assessment (○).

Glucose disposal rates (GDR) were calculated prior to and at the completion of the programme and a significant overall trend was observed with BMI (Figure 4) whereby the higher BMI was associated with a lower GDR (*r* = 0.58, *p* = 0.006).

## Discussion

After an 8-week moderate intensity exercise programme women with PCOS had significantly decreased levels of CD105+ MP and improved GDR. This may indicate some improvement in cellular health as a result of a short-term, supervised aerobic exercise programme, independent of dietary modifications. An additional finding of this study was that PCOS women displayed significantly higher CD105+ MP at baseline when compared to otherwise healthy, non-PCOS females (Table 1). CD105+ MP may be expected to be higher and associated with poor endothelial function as CD105 (Endoglin) is a constitutively expressed protein on the surface of all endothelial cells and therefore would be expected to be present on all EMP released whereas VCAM-1 (CD106) is a marker of activation. The data suggests that there is no difference between the groups in terms of endothelial cell activation but there is a significantly higher amount of circulating CD105+ MP in the PCOS group. The decrease in circulating CD105+ MP may be indicative of an improvement in endothelial function. Previously Miniello et al. (17) demonstrated a link between insulin resistance and endothelial function and from our data here an improvement in GDR was accompanied by a decrease in circulating CD105+ MP.

This finding demonstrates that CD105+ MP may be a useful marker of endothelial function, as others have also shown that women with PCOS have endothelial dysfunction, using a variety of different assessment methods, such as FMD (10, 15, 34) and endothelial adhesion molecules (10). Furthermore, women with PCOS significantly improved their metabolic risk, including body mass, SBP, DBP, aerobic fitness, BMI and WC by the end of the programme (Table 2).

The reduction in circulating CD105+ MP reported here for the PCOS women may indicate accord with a study by Sprung et al. (14) who found that EF (BA-FMD) was improved in women with PCOS when compared to control women with PCOS who received conventional care following a 16-week aerobic exercise intervention. Ten women with PCOS underwent supervised 30 min exercise 3 times a week at 30% heart rate reserve (HRR), which progressed weekly based on HR responses (up to 60% HRR). The control group received lifestyle advice from clinical consultations prompting them to lose weight and increase their physical activity. Various assessments were measured during the study, including BA-FMD, cardiorespiratory fitness, visceral and abdominal subcutaneous adipose tissue, glycaemic control, and lipid and hormone profiles. When comparing the 2 groups, BA-FMD and cardiorespiratory fitness improved from pre to post-exercise intervention in the exercising group only, suggesting that supervised aerobic exercise in women with PCOS can enhance EF. Sprung et al. (14) however did not use women without PCOS as controls, as it would also have also been useful to see any differences between PCOS women and control women without PCOS following the two interventions. Baseline body composition data was similar between the two studies [BMI was 31.1 kg^.^m^2^ in this study compared to 31 kg^.^m^2^ in (14); WC was 98.05 and 100 cm, respectively, whilst body mass was 85.45 kg compared to 82.1 kg). However, despite not being statistically significant there was an observed difference between body mass and BMI reported here between the two groups, with the PCOS group being higher in both assessments (Table 1). Also there is potential for medication to impact EF within the PCOS group however participants taking any oral anti-diabetic medicine to insulin sensitivity or weight reduction medication were excluded from the study. Comparatively to these findings, Roessler et al. (35) investigated the effects of exercise and group counseling on body composition and aerobic fitness in 17 women with PCOS. WC, BMI, body mass and VO_2max_ were improved in PCOS women following a cross-over designed trial with the exercise programme consisting of 3 sessions per week with 2 days of indoor cycling and 1 day of brisk walking/running.

The exercise in Sprung et al. (14) was moderate in intensity, although they used %HRR (30% HRR initially, rising to 60% HRR) instead of %VO_2max_ as in the present study (60% VO_2max_). Initially, exercise was performed 3 times a week, as in the present study, but for a period of just 30 min, which was then increased to 5 times per week up to 45 min per session, whereas a fixed protocol was adhered to in the present study. Nonetheless, Sprung et al. (14) increased the length of their sessions and frequency of training per week, presumably as increases in fitness were observed in each group, similar to our reassessing their mid-point fitness so that improvements in each individual's fitness could be used to re-evaluate the intensity for the second half of the programme. These studies suggest that supervised moderate aerobic exercise of 30–60 min 3–5 days a week is sufficient to improve EF and other CV risk factors, such as WC, body mass and BP; however, changes in these parameters did not correlate with EMP/EF. The changes in body mass seen in the PCOS group were modest but significant from pre to post-exercise intervention in the current study (85.45–84.04 kg), suggesting that improvements in cardiorespiratory fitness without substantial weight loss may reduce the CV risk in women with PCOS, a finding also reported by others (14, 36).

Research assessing EF following exercise interventions in PCOS women by endothelial biomarkers is very limited (14). However, one study (18) looked at the effects of exercise training on EF in PCOS women measuring circulating biomarkers through a multiplex analyser (sCD106, sICAM-1) and plasminogen activator inhibitor-1 (PAI-1). This study used 3 different groups, one group was a controlled diet with moderate aerobic exercise (60–80% HR_max_), one used diet combined with aerobic-resistance training, and the other a diet only without exercise. sCD106, sICAM-1, and PAI-1 all reduced at week 20 in each group compared to pre exercise programme, but the parameters did not differ between groups.

Previous studies have shown that there was a significant impairment in EF in PCOS women at baseline levels compared to control women without PCOS (37–39). El-Kannishy et al. (12) found that EF, as assessed by BA-FMD, was impaired in both lean and obese women with PCOS. They assessed 22 obese women with PCOS as well as 14 lean women with PCOS. Flow-mediated dilation was significantly decreased in both groups of women with PCOS. Further to this, it was found that FMD was not correlated with BMI or IR. These findings suggest that women with PCOS who are otherwise free of any other known disease associated with PCOS still show characteristics of ED, and therefore both lean and obese participants could still benefit from an exercise intervention similar to that used in this study to improve EF, even in the absence of other risk factors.

A review of PCOS and exercise highlighted the various limitations that are common in exercise interventions in women with PCOS (2). Studies have generally been small in sample size and have had a significant drop out rate (2). Based on the verbal feedback provided throughout the study period, this high compliance reported here was mainly due to the supervision and monitoring of the exercise sessions, as well as the duration and intensity of exercise sessions being favorable to participants, and participants recruited were also highly motivated and volunteered to take part. This leads to the suggestion perhaps that a home based programme, at least in the early stages of an intervention, may not be the best route to take, and that closely monitored, supervised exercise interventions appear to have a positive effect. Additionally, the effects of group based exercise warrants future investigation given that social interaction may be an exercise motive for some individuals to enable the social element of group training. A further issue with existing literature is that studies tend to use young women who were predominantly obese (2). This was one of the reasons for the inclusion of lean women as well as obese in this study.

This study confirms that moderate intensity exercise (60% VO_2max_) could be recommended for this population. The data here also suggests that this type of moderate intensity exercise is sufficient over a relatively short time frame to see improvements in anthropometric measurements and a reduction in circulating EMP. This is in agreement with other studies who have also found improvements in women with PCOS as a result of moderate intensity exercise (18, 40) but future work could also investigate the use of incorporating high intensity interval training or resistance training into an exercise/diet programme in PCOS women which has previously shown favorable results in body composition and aerobic fitness (35).

There are several pharmaceutical interventions for PCOS, which primarily focus on addressing reproductive dysfunction and IR (2). However, lifestyle modification remains the first line management to improve cardiovascular and reproductive risk factors in women with PCOS (2, 41). The presented research strengthens the idea that exercise should remain one of the first line treatments for women with PCOS. EMP can be significantly reduced following the prescription of moderate aerobic exercise training, independent of any dietary restrictions.

## Conclusion

CD105+ MP may be utilized as a circulating biomarker in women with PCOS and a reduction in numbers following an exercise programme is suggestive of an improvement in endothelial cell health.

## Data Availability

The datasets generated for this study are available on request to the corresponding author.

## Author Contributions

LM, SA, and RV designed the study. RK, DP, and MA collected and analyzed the data. All authors contributed to writing the manuscript and final approval.

### Conflict of Interest Statement

The authors declare that the research was conducted in the absence of any commercial or financial relationships that could be construed as a potential conflict of interest.
